# Evaluation of the Appropriate LigaSure™ Device to Transect the Appendix—A Comparison between 5 mm and 10 mm Laparoscopic Devices in an Ex Vivo Trial

**DOI:** 10.3390/medicina59050927

**Published:** 2023-05-11

**Authors:** Salmai Turial, Martin Schwind, Alexandra Nyiredi

**Affiliations:** 1 Department of Pediatric Surgery, Pediatric Trauma and Pediatric Urology, University Medical Centre Magdeburg, 39120 Magdeburg, Germany; 2 Department of Pediatric Surgery, University Medical Centre Mainz, 55101 Mainz, Germany

**Keywords:** appendectomy, LigaSure™, laparoscopy

## Abstract

*Background and Objectives*: A topic of greatinterest in the surgical field comprises cost and time reduction operative techniques with high efficiency rates. Thus, the aim of this paper is to evaluate whether a transection of the appendix using only a laparoscopic LigaSure™ device is feasible and, if so, which size of the laparoscopic device is optimal. *Materials and Methods*: Appendectomy specimens were sealed and cut using LigaSure^TM^ V (5 mm) and LigaSure Atlas^TM^ (10 mm) devices ex vivo. Analysis criteria included handling, resistance to bursting pressure of the appendicular stump (adequacy), eligibility, durability and airtightness. *Results*: Twenty sealed areas were measured. While the 5 mm instrument was not able to transect the appendix in one attempt in any of the cases, the 10 mm device could be applied successfully without any handling difficulties. The adequacy of the sealed area was rated as complete and dry in all 10 cases using the 10 mm device and as oozing in 8 of the cases using the 5 mm device. There was no leakage in terms of air and liquid tightness using the 10 mm device, in contrast to six sealed segments with air and liquid leakage when using the 5 mm device. The resistance to bursting pressure was on average 285 mmHg and 60.5 mmHg with the 10 mm and 5 mm devices, respectively. The durability and eligibility of the 10 mm device were rated as very sufficient in 9 of 10 cases (1 perforation) in contrast to the 5 mm device, where the sealing in 9 of 10 cases was not sufficient (9 perforations). *Conclusions*: Using the 10 mm laparoscopic LigaSure™ device for the transection of the appendix seems to be feasible, safe and resistant to 300 mmHg bursting pressure. The 5 mm LigaSure™ instrument is inadequate to seal the appendix in humans.

## 1. Introduction

One of the most frequent causes of acute abdominal pain in children is acute appendicitis, which in most cases necessitates a surgical resolution that can be either open or laparoscopic [1].

Since 1983, when it was first described, laparoscopic appendectomy has grown to be the procedure of choice for both simple and complicated appendicitis [2,3] due to lower rates of postoperative morbidity and shorter hospital stay than open surgery [4,5,6,7,8]. In laparoscopic appendectomy, a variety of techniques are frequently used to close the appendiceal stumps. Among them are the endostapler, loop ligature and locking polymeric clip. Due to their affordability and simplicity of use, locking polymeric clip and the loop ligature are the most frequently employed techniques for appendiceal stump closure. Up-to-date studies on the finest methods for mesoappendix ligation and stump closure throughout laparoscopic appendectomy are still controversial, and surgeons tend to favor one method over another depending on things such as training, cost, availability and time commitment [9,10,11].

The mesoappendix division can also be accomplished using either mechanical or simple electrocautery methods. Plentiful new vessel-sealing utensils are used in surgery and are gaining popularity, such as electrothermal bipolar-activated LigaSure and Harmonic’s ultrasonic system [12,13].

The LigaSure Vessel Sealing System (LVSS) is a bipolar electrosurgical device that creates bipolar hemostasis by denaturing collagen, elastin and connective tissues. The LVSS actually facilitates tissue fusion creating a true seal, in contrast to simple bipolar cautery. The LVSS uses a feedback-controlled response that automatically interrupts the energy delivery when the seal cycle is complete [14,15]. The LVSS has been used to secure hemostasis in various open and laparoscopic procedures in recent decades [16,17,18,19,20,21]. Even though the LVSS was originally designed to seal vessels, its use in the dissection/transection of various soft and parenchymatous tissues has also been reported progressively more often in recent literature [22,23,24,25,26,27,28,29,30,31,32,33].

In the last ten years, Turial et al. [34] have had a good experience using the LVSS in the closure of the cystic duct during laparoscopic cholecystectomies. Additionally, the use of the LVSS in the division of the mesoappendix during laparoscopic appendectomy has been reported by both Aydogan et al. [35] and Sucullu et al. [36] in a series of 127 and 32 cases, respectively, with good results. Transection of the apex ceaci is mainly reported in animal models [37]; conversely, Gupta et al. [38] reported a series of 53 patients with successful appendectomies using a 5 mm LigaSure^TM^ instrument.

Our goal was to consider the use of the LVSS for appendix transections in human patients and define its limits. Therefore, we aimed to evaluate the bursting pressure (BP) limits in sealed appendix specimens by comparing the use of 5 mm and 10 mm laparoscopic LigaSure^TM^ devices.

## 2. Materials and Methods

An ex vivo trial was conducted on appendectomy specimens harvested after routine laparoscopic appendectomies due to acute appendicitis with no local complications. The diagnosis of acute appendicitis and indication for laparoscopic appendectomy was based on routine clinical and ultrasonographic criteria unrelated to the present study.

The laparoscopic appendectomy was performed routinely through loop ligature. The appendectomy was performed using two-loop ligatures. The appendix was transected between the two-loop ligatures; one remained on the appendix base in vivo, and the other one was removed with the appendix specimen.

Written consent was obtained from all patients to use the appendix specimens harvested after an appendectomy. The appropriate governmental medical ethics committee (Landesärztekammer Rheinland-Pfalz, 837.456.10 (7465)) approved the study.

**Figure 1:**
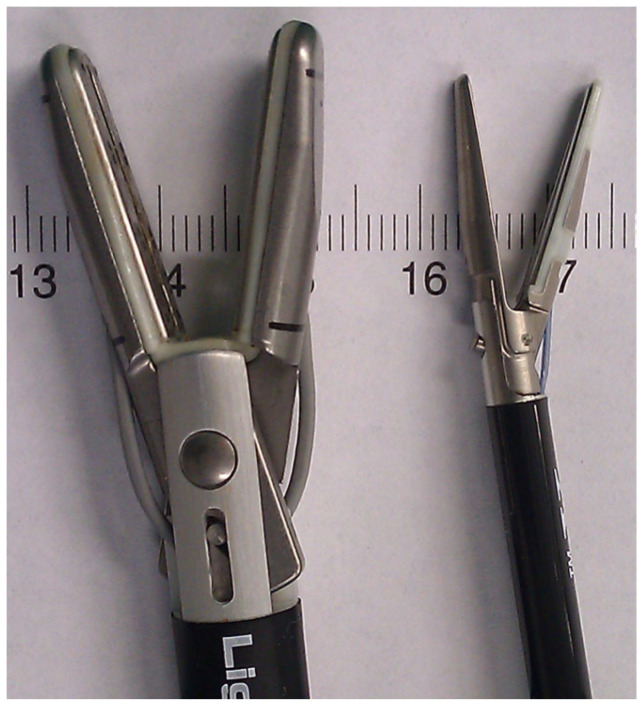
Image demonstrating the jaw dimensions of the 5 mm and 10 mm laparoscopic LigaSure^TM^ devices.

The appendix specimens were ex vivo sealed and cut using laparoscopic LigaSure^TM^ devices. We used and compared two laparoscopic LigaSure^TM^ devices: LigaSure^TM^ V and LigaSureAtlas^TM^ (Covidien-Valleylab, Boulder, CO, USA). The LigaSureAtlas^TM^ hand-switching device (Figure 1) has a shaft diameter of 10 mm, a seal length of 22 mm, a seal width of 6 mm (2 × 3 mm) and a cut length of 20 mm. The LigaSure^TM^ V 5 mm device has a shaft diameter of 5 mm, a seal length of 18 mm, a seal width of 4.6 mm (2 × 2.3 mm) and a cut length of 12 mm.

The appendix specimens included in the study had a minimum length of 4 cm, and the maximum accepted inflammation grade was Phlegmonous, according to the intraoperative findings, which were later confirmed by the histopathological findings and described as Ulcerophlegmonous appendicitis, with explicit mention of the absence of perforation in all selected cases. Specimens from patients with macroscopic perforated appendicitis and those showing post-chronic inflammatory changes were excluded from the study because these inflammatory changes could negatively impact the bursting pressure test.

Each appendix specimen was sealed twice with a 5 mm and a 10 mm device. There was a 1 cm gap between the two sealed areas. All procedures were conducted on the proximal two-thirds of the specimens; nonetheless, the tip of the appendix specimen was not subjected to the sealing and cutting process. In this manner, both instruments were used on a similar specimen diameter. The adequacy of the cutting and sealing process and bursting pressure of the sealed appendix segment were compared between the two LigaSure^TM^ devices, 5 and 10 mm. The handling of the specimens was performed immediately after the operation on the patient ended, in the same operating theatre. The specimens were handled between 15 and 25 min after extraction and were not submerged in any chemical substances; thus, no changes could be encountered due to mechanical or chemical factors. After the experimental procedure ended, the specimens were processed in a standard manner. The specimens were fixed in formaldehyde and sent for histopathological examination.

All experimental procedures were sequentially photo- and film-documented. The footage was later used for the analysis of the results.

The current research aimed at meeting primary and secondary criteria as follows: the primary criteria were handling and resistance to bursting pressure (expressed in mmHg) of the sealed appendicular stump. Handling was defined and expressed as the number of attempts to successfully seal the appendicular lumen. The sufficiency of the sealing process was expressed as either (1) an oozing seal that required additional intervention or (2) a complete and dry seal.

The secondary criteria were eligibility, durability, air and liquid tightness and the integrity of the sealed area. The time needed to complete the sealing process was not considered in this trial because it is not clinically relevant and most likely depends on the mass and rigidity of the sealed tissue (feedback-controlled response of the device). The sealed appendix segments were placed in saline liquid and air, and a diluted povidone-iodine solution was pumped through a 26 G injection needle to detect air leakage.

**Figure 2:**
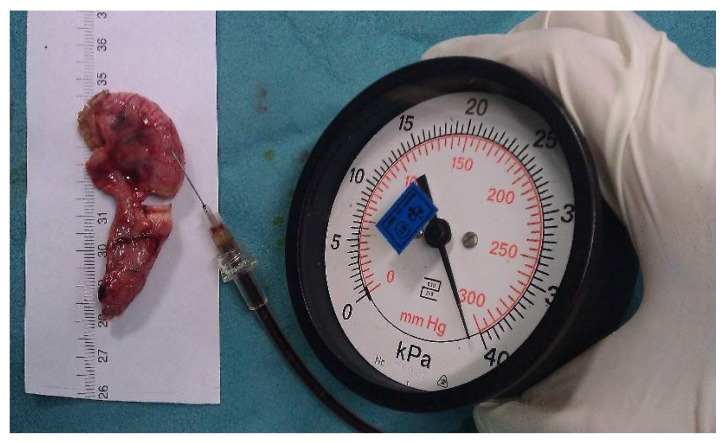
Image demonstrating the bursting pressure measurement.

Bursting pressure measurements: To measure the bursting pressure, a standard mercury manometer (Hellmeyer, Sankt Augustin, Germany) was connected to a three-way cock of the infusion line. The other two valves of the three-way cock were connected to a syringe and a 26 G injection needle. The syringe was filled with a diluted povidone-iodine solution to improve visualization. The injection needle was inserted intraluminally into each appendix stump. The pressure was recorded (mmHg) as the peak pressure attained before the rupture of the sealed area. If no rupture occurred, the maximum pressure of 300 mmHg (the maximum on the manometer’s scale) was recorded (Figure 2).

The results were statistically processed using SPSS 29. Since the two instruments were used on the same appendices, they are paired samples. Accordingly, tests for paired samples were used.

With respect to the qualitative variables—adequacy, leakage and histopathological finding of the sealed area—McNemar tests would be used for the corresponding comparisons. However, these require that the number of rows and columns match, which is not the case because only one value (dry or no perforation) occurs in each case for the 10 mm instrument. Therefore, nonparametric tests were used for all variables. The significance level is considered to be 0.05 (CI 95%).

## 3. Results

Ten appendix specimens were used for the transection of the appendix using both the 5 mm and the 10 mm LigaSure^TM^ devices. This resulted in a total of twenty sealing areas. The diameter of the appendices was on average 12 mm (range: 9–16 mm).

**Figure 3:**
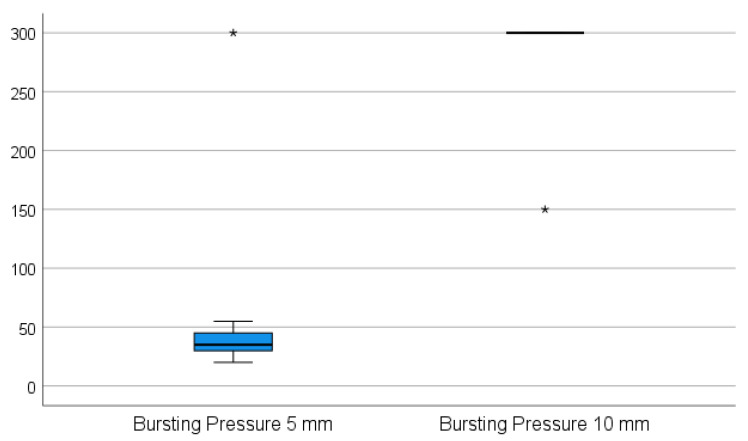
The average bursting pressure for the 5 mm device vs. the 10 mm device. (* The maximum pressure applied to produce a perforation in the sealed appendix specimen—300 mmHg for the 10 mm LigaSure^TM^ instrument, respectively 150 mmHg for the 5 mm LigaSure^TM^).

While the 5 mm instrument was not able to transect the appendix in one attempt in any of the cases, the 10 mm device was applied successfully in one attempt without any handling difficulties. When using the 5 mm device, it was not possible to properly grasp the appendix during the sealing procedure since the appendix slipped out of the jaws of the instrument when the tissue was sealed and subsequently shrunk. The 5 mm device was repeatedly applied on the stump, on average three times (*p* = 0.002). The appendix specimens shrunk and were partially sealed. The sealed part was then cut for a better further grasp on the remaining part of the specimen, and the process was repeated until the specimen was transected. All of the stumps were completely sealed in one application using the 10 mm device. The adequacy of the sealed area was rated as complete and dry in all ten cases using the 10 mm device and as oozing in eight of the cases using the 5 mm device (*p* = 0.008). There was no leakage in terms of air and liquid tightness in all cases when using the 10 mm device, in contrast to six sealed segments with air and liquid leakage when using the 5 mm device (*p* = 0.031). The resistance to bursting pressure was on average 285 mmHg with the 10 mm device. In nine of these cases, the maximum measurable pressure on the manometer used (300 mmHg) was achieved without any sign of perforation. In these instances, the manometer was removed from the valve, and approximately twice as much pressure was applied by hand, still resulting in no sign of perforation. In only one case in this group was extravasation of the fluid recorded at 150 mmHg on the very edge of the sealed area; however, in this case, the device was not applied appropriately (the appendix specimen was clamped between the jaws too tangentially). The average bursting pressure for the 5 mm device was 60.5 mmHg, and in nine cases, a perforation was noted (*p* = 0.004) as shown in Figure 3. There was no perforation recorded at a maximum pressure of 300 mmHg in only one case. All of the perforations occurred in places between the sealed areas where sealing had previously been performed in multiple steps. 

All these results were further confirmed by the histopathological examination (*p* = 0.008), namely, the inflammation level of the appendix specimen as Ulcerophlegmonous with no perforation and also the seal status for each of both instruments. Statistical results and frequency information are shown in Table 1 and Table 2, respectively.

**Figure 4:**
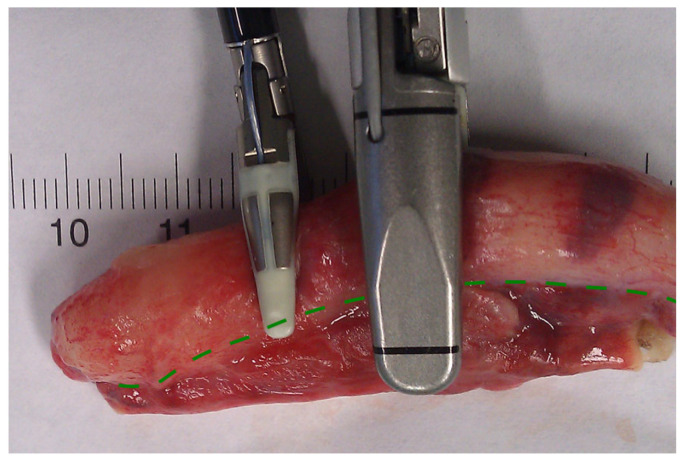
Appendix specimen between the jaws of the LigaSure^TM^ devices.

**Figure 5:**
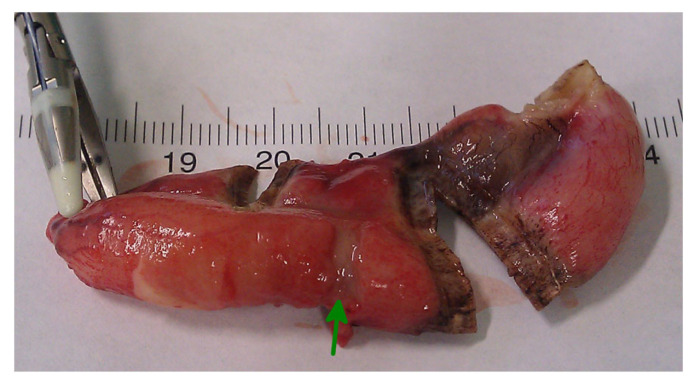
Transection surface after application of the Ligasure^TM^ devices.

**Figure 6:**
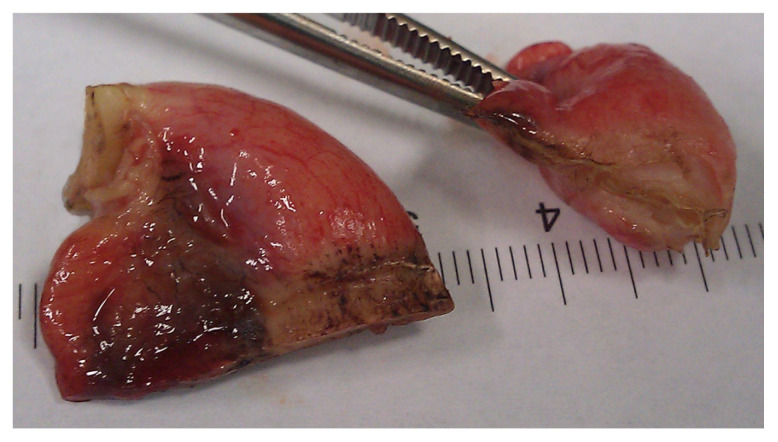
Transection surfaces after complete Ligasure application using the 10 mm device (left) and the 5 mm device (right).

In conclusion, based on the results measured above, the durability and eligibility of the 10 mm device were rated as very sufficient in nine of ten cases in contrast to the 5 mm device, where the sealing in nine of ten cases was not sufficient. When using both the 5 and 10 mm instruments, it became clear that the 10 mm device has substantially more power to compress the appendix tissue between the jaws. In contrast, the 5 mm device jaw angle (“dolphin nose”, 2.6 mm at the tip) was unable to hold the appendix tissue with high consistency, as shown in Figure 4, Figure 5 and Figure 6. In Figure 4, the specimen is held with no compression on the appendix. The 10 mm device covers the whole appendix lumen, including the mesoappendix, and the 5 mm device does not. In Figure 5, the appendix and a large part of the mesoappendix were divided in one attempt by the 10 mm device. Using the 5 mm device, it was not possible to transect the appendix from its antimesenterial surface in one attempt. An arrow indicates the site where compression of the appendix was attempted by the 5 mm device. The 5 mm device in this figure was therefore used on the mesoappendix to demonstrate the sealed surface of the same specimen. In Figure 6, the sealed surfaces using the 10 mm and 5 mm instruments are shown. In Figure 6 (left), the sealed surface, achieved in only one application of the 10 mm instrument, resulted in a completely sealed area in one line. Figure 6 (right) shows the sealed surface after the application of the 5 mm instrument in four steps and the resulting eroded transection surface.

Due to the clear results and the design of the study, further statistical analysis or histological examination of the specimens were deemed unnecessary in the present report.

## 4. Discussion

In the present study, we observed that the use of the LVSS for appendix transections in human appendix specimens is feasible for the 10 mm device. The diameter of the appendix specimens was 12 mm on average (range: 9–16 mm). Searle et al. [39] published a complex work stratifying the diameter and length of the normal appendix in the pediatric population. They found that mean diameters were as follows: 3.7 (±1.3) mm for children under the age of 3, 6.3 (±1.2) mm for children between the age of 3 and 9, 6.7 (±1.6) mm for children aged between 9 and 13 and 6.9 (±1.6) for children of and over 13 years of age. Furthermore, the literature states that the 6 mm diameter is a cutoff for appendicitis [40]. Considering both the above-mentioned studies and our findings, the appendicular stump varies in diameter with both age and disease severity. The absence of a thorough study regarding the age stratification of the diameter and length of the appendix in acute appendicitis in the pediatric population coupled with the 6 mm cutoff for acute appendicitis questioned by Trout et al. [40] shows that a preoperative preselection of the device diameter is not feasible. 

The use of various energy-based sources has become even more popular in laparoscopic surgery, as they are easy to apply intracorporeally compared to traditional surgical techniques, e.g., ligatures, suturing and tying [17,18,20,21,26,27,28,29,30,41,42]. The LVSS was originally designed for sealing vessels as an alternative to the use of clips or ligature. Comparative studies have proven that it is as safe, feasible and even as beneficial as other vessel closure techniques, such as the plasma trisector, surgical clip application, harmonic scalpel and conventional hemostasis [17,18,21,29,30,32,33]. Over time, the use of the LVSS for dissection and transection of various soft and parenchymatous tissues (such as during a liver resection, pancreatectomy or hysterectomy) [16,17,18,19,20,21,22,23,24,25,26,27,28,29,30,31,32,33,34] has been increasingly prevalent in the literature in addition to reports on its use in sealing vessels.

In contrast to many reports about its use for vascular, parenchymatos or intestinal tissues in clinical or animal trials, there are only a few reports about its use during an appendectomy.

Two reports from Turkey in 2009 [35,36], including a total of 143 patients, confirmed the beneficial efficacy of the ligature devices for the division of the mesoappendix during laparoscopic appendectomies compared to the use of an endodissector and endoclip. According to these reports, the division of the mesoappendixcan be performed with a stepwise application that makes it possible to divide the mesoappendix rapidly and without bleeding using the LVSS.

In the report by Sucullu et al. [36], a 5 mm or a 10 mm laparoscopic LigaSureTM instrument was used, whereas in the report by Aydogan et al. [35], a 10 mm device (LigaSureAtlas^TM^) was used. Unfortunately, neither paper reported details on the differences in handling during the transection of the mesoappendix.

Others report [43] good results using electrocautery alone to divide the mesoappendix, emphasizing its cost effectiveness compared to more expensive instruments used for the same purpose, such as the endostapler, LigaSure^TM^ or the harmonic scalpel.

The results of the current report on the transection of the appendix show that the 10 mm laparoscopic device is superior to the 5 mm laparoscopic instrument. The need for stepwise sealing and transection of the appendix using a 5 mm device resulted in a high number of incomplete sealed gaps with a consequently higher number of perforations at a low bursting pressure level. The histopathological findings supported our clinical findings, namely, that the stepwise seal of the appendix specimen using the 5 mm LigaSureTm device produced an insufficient seal, thus leaving gaps prone to leakage and perforation at low bursting pressure.

Burst pressure has proven effective in evaluating intestinal anastomotic sufficiency, particularly in terms of mechanical stability. Therefore, we measured the bursting pressure of the appendix specimen to assess the sealing adequacy of the LigaSure^TM^ device.

Only one paper was found in a search using the PubMed database (including words appendectomy and LigaSure^TM^) that compared the LigaSure^TM^ device to the absorbable ligatures and endoclips in transecting the appendix in animal investigations [37]. The paper found that better healing, less inflammation, shorter operation time and equal strength were achieved with the LigaSure^TM^ device compared to the polyglactin 910 ties and endoclips in an experimental appendectomy. However, two concerns were reported that are not relevant to the use of LigaSure^TM^. The animal model chosen for this trial was a Sprague–Dawley rat, in which an appendix vermiformis, or a cecal appendage as in apes and humans, is absent. With regard to the figures published in this paper, the term coecotomy is more appropriate than appendectomy. In our opinion, the investigators performed an amputation of the apex caeci in the rats (Rodentia), as the rats possess a so-called cecum amplium. Rabbits (Lagomorpha), for example, possess an appendix vermiformis. The authors clarified the “appendix-like structure” transected in their trial.

Gupta et al. [38] reported a series of 53 patients aged between 11 and 60 years with a successful appendectomy using a 5 mm LigaSure^TM^ instrument that was repeatedly applied to obtain a resection. This also supports our findings, namely, that the 5 mm instrument achieves resection after repeated application. The author reported two complications that were unrelated to appendectomy: one case of mild subcutaneous emphysema over the abdomen that was managed conservatively and a postoperative surgical site infection that was managed without reoperation. The 5 mm instrument was considered inefficient for this study, as a repeated application of the device was needed on the appendix specimen through a sealing–cut–sealing manner so that the tissue could be further grasped, which made the specimen shrink and alter its structure, thus making it susceptible for leakage/spillage.

Another topic of great interest is cosmetics, particularly for pediatric patients. This makes selecting an instrument’s diameter more challenging because the instrument must be adapted for the patient and the particular clinical setting. Therefore, it is true that smaller ports are needed. However, our research demonstrates that the 5 mm device we used cannot provide the required sealing. What is more, the 10 mm instrument was used on the umbilical site. The umbilical operative site was routinely used for the extraction of the appendix specimen after a laparoscopic appendectomy due to better cosmetics. Therefore, the use of a 10 mm instrument on the umbilical site does not negatively impact cosmetic outcomes. Furthermore, according to recent studies on the mechanism of bowel movement and its pathologies, it was shown that the normal pressure in the cecum is 60–80 mmHg, which decreases to 20–40 mmHg in the central part of the large bowel and then increases to 60–100 mmHg in the proximity of the rectum [44,45,46,47,48]. This finding supports our theory that a maximum burst resistance of 60.5 mmHg achieved with a 5 mm instrument is insufficient.

Mannu et al. [9] state that the method of choice to close the appendix stump does not significantly impact the operative costs. However, a new approach should be considered with the addition of sealing and appendectomy using a LigaSure^TM^ device. Further research is needed in this field to determine whether time savings and safer closure outweigh the higher costs of mechanical devices.

The findings of this study have to be seen in the light of some limitations. The small number of specimens included in the study may reveal a number of false positive results. On the other hand, the absence of a control group with histopathologically unmodified appendix specimens gives bias in the interpretation of the results. However, a comparison group of human appendix specimens may not be achieved, as the indication for appendectomy in the absence of acute appendicitis is scarce and extremely limited. Bias can also be avoided by having a database of appendix stumps characteristics that is age- and disease-severity-oriented. Finally, the relation of the sealing instrument to the appendix base must be considered. The appendix base is naturally smaller in diameter than the corpus of an inflamed appendix.

## 5. Conclusions

In our investigation, we found impressive differences between the 5 mm and 10 mm LigaSure^TM^ instruments used for appendectomy specimens in terms of handling and adequacy of the appendix transection. Even though the specific parameters of the jaws of the 5 mm and 10 mm devices differ by only a few millimeters (5 mm/10 mm instrument seal width: 4.6 mm as compared to 6 mm, seal length: 18 mm/as compared to 22 mm and cut length: 12 mm as compared to 20 mm), it seems that the resulting force to press the tissue between the jaws in the 10 mm instrument is significantly stronger than the 5 mm instrument. This difference is more evident when used on appendix tissue with high consistency. As the use of the 10 mm LigaSure^TM^ device to transect an inflamed appendix seems to be highly satisfactory, we began to use this device in a clinical trial. The results of this case series will be reported in the near future.

## Figures and Tables

**Table 1:** Statistics tests.

Statistics Test ^a^		
	Exact sig. (2-tailed)	Exact sig. (1-tailed)
Number of grasps with the 10 mm instrument vs. grasps with the 5 mm instrument	0.002 ^b^	<0.001
BP with the 10 mm instrument vs. BP with the 5 mm instrument	0.004 ^b^	0.002
Adequacy with the 10 mm instrument vs. Adequacy with the 5 mm instrument	0.008 ^b^	0.004
Leakage with the 10 mm instrument vs. Leakage with the 5 mm instrument	0.031 ^b^	0.016
Histopathological finding of the sealed area (HPFSA) with the 10 mm instrument vs. Histopathological finding of the sealed area with the 5 mm instrument	0.008 ^b^	0.004

^a^ sign test; ^b^ Used binomial distribution.

**Table 2:** Frequencies.

		N	
Grasps 10 mm instrument vs. Grasps 5 mm instrument	Negative Differences	10	Grasps 10 mm < Grasps 5 mm
	Positive Differences	0	Grasps 10 mm > Grasps 5 mm
	Ties	0	Grasps 10 mm = Grasps 5 mm
	Total	10	
BP 10 mm instrument vs. BP 5 mm instrument	Negative Differences	0	BP 10 mm < BP 5 mm
	Positive Differences	9	BP 10 mm > BP 5 mm
	Ties	1	B Press 10 mm = B Press 5 mm
	Total	10	
Adequacy 10 mm instrument vs. Adequacy 5 mm instrument	Negative Differences	8	Adequacy 10 mm < Adequacy 5 mm
	Positive Differences	0	Adequacy 10 mm > Adequacy 5 mm
	Ties	2	Adequacy 10 mm = Adequacy 5 mm
	Total	10	
Leakage 10 mm instrument vs. Leakage 5 mm instrument	Negative Differences	6	Leakage 10 mm < Leakage 5 mm
	Positive Differences	0	Leakage 10 mm > Leakage 5 mm
	Ties	4	Leakage 10 mm = Leakage 5 mm
	Total	10	
HPFSA 10 mm–HPFSA 5 mm	Negative Differences	8	HPFSA 10 mm < HPFSA 5 mm
	Positive Differences	0	HPFSA 10 mm > HPFSA 5 mm
	Ties	2	HPFSA 10 mm = HPFSA 5 mm
	Total	10	

## Data Availability

No new data were created or analyzed in this study. Data sharing is not applicable to this article.
